# Safety and Efficacy of miltefosine alone and in combination with sodium stibogluconate and liposomal amphotericin B for the treatment of primary visceral leishmaniasis in East Africa: study protocol for a randomized controlled trial

**DOI:** 10.1186/1745-6215-12-166

**Published:** 2011-06-30

**Authors:** Raymond Omollo, Neal Alexander, Tansy Edwards, Eltahir AG Khalil, Brima M Younis, Abuzaid A Abuzaid, Monique Wasunna, Njenga Njoroge, Dedan Kinoti, George Kirigi, Thomas PC Dorlo, Sally Ellis, Manica Balasegaram, Ahmed M Musa

**Affiliations:** 1 Drugs for Neglected Diseases initiative (DNDi) Africa, Centre for Clinical Research, Kenya Medical Research Institute, Nairobi, Kenya; 2 MRC Tropical Epidemiology Group, London School of Hygiene and Tropical Medicine, London, UK; 3 Institute of Endemic Diseases, University of Khartoum, Khartoum, Sudan; 4 Centre for Clinical Research, Kenya Medical Research Institute, Nairobi, Kenya; 5 Division of Infectious Diseases, Tropical Medicine & AIDS, Academic Medical Center, Amsterdam, the Netherlands; 6 Department of Pharmacy & Pharmacology, Slotervaart Hospital, Amsterdam, the Netherlands; 7 DNDi, Geneva, Switzerland

**Keywords:** Visceral Leishmaniasis, Miltefosine, AmBisome^®^, Tri-angular test, protocol, RCT

## Abstract

**Background:**

Treatment options for Visceral Leishmaniasis (VL) in East Africa are far from satisfactory due to cost, toxicity, prolonged treatment duration or emergence of parasite resistance. Hence there is a need to explore alternative treatment protocols such as miltefosine alone or in combinations including miltefosine, sodium stibogluconate (SSG) or liposomal amphotericin B. The aim of this trial is to identify regimen(s) which are sufficiently promising for future trials in East Africa.

**Methods/Design:**

A phase II randomized, parallel arm, open-labelled trial is being conducted to assess the efficacy of each of the three regimens: liposomal amphotericin B with SSG, Liposomal amphotericin B with miltefosine and miltefosine alone. The primary endpoint is cure at day 28 with secondary endpoint at day 210 (6 months). Initial cure is a single composite measure based on parasitologic evaluation (bone marrow, spleen or lymph node aspirate) and clinical assessment. Repeated interim analyses have been planned after recruitment of 15 patients in each arm with a maximum sample size of 63 for each. These will follow group-sequential methods (the triangular test) to identify when a regimen is inadequate (<75% efficacy) or adequate (>90% efficacy). We describe a method to ensure consistency of the sequential analysis of day 28 cure with the non-sequential analysis of day 210 cure.

**Discussion:**

A regimen with adequate efficacy would be a candidate for treatment of VL with reasonable costs. The design allows repeated testing throughout the trial recruitment period while maintaining good statistical properties (Type I & II error rates) and reducing the expected sample sizes.

**Trial Registration:**

ClinicalTrials.gov: NCT01067443

## Background

Visceral leishmaniasis (VL), or kala-azar, is the most serious of all Leishmania infections and is fatal if left untreated [1]. A complication of VL, particularly in Sudan, is post kala-azar dermal leishmaniasis (PKDL) where patients have no detectable parasites in the spleen, bone marrow or lymph nodes but develop macular and papulomacular skin lesions in which leishmania parasites may be detected. The median time to occurrence of PKDL is 6 months after treatment [2].

The monotherapeutic treatment options available in East Africa are far from satisfactory due to cost (Liposomal amphotericin B^®^), toxicity (sodium stibogluconate, SSG) or prolonged treatment duration (one month, SSG), resulting in the additional concern of compliance and possible emergence of parasite resistance. Paramomycin, both in monotherapy and in combination with SSG has just recently completed development in the East Africa. However its use in monotherapy does not appear realistic in the region [3,4]. This calls for exploration of new treatment options including miltefosine monotherapy and alternative combination treatment algorithms combining drugs with different modes of action like SSG, Liposomal amphotericin B^® ^and miltefosine which could provide new treatment option with reasonable costs due to reduction in needed dosages and treatment duration and less risk of resistance emerging [5].

Sodium stibogluconate for 30 days is currently the mainstay of VL treatment in East Africa. Liposomal amphotericin B^® ^has also been used in the field, but primarily as a rescue treatment for VL. There is limited experience of the use of miltefosine which is not registered in the region: data are available from only one study, conducted in Ethiopia, which demonstrated efficacy of 93% for miltefosine monotherapy at 6 months follow-up in HIV negative patients [6]. All three drugs have been studied and are registered in India and a phase IV study for miltefosine (28 day course) has been completed [7,8]. In addition a phase-III study conducted in India evaluated 3 short course combinations, including Ambisome plus miltefosine for 7 days, which had a 6 month efficacy of 97% [9].

This study intends to look at potential feasible short course combination therapies as well as evaluate and possibly register miltefosine in its conventional dose and regimen for VL in Sudan and Kenya. It is also intended to supply miltefosine pharmacokinetics in VL patients in East Africa, including children, for whom there is little such information globally. It is a phase-II proof of concept study for efficacy and safety of two potential combinations.

## Methods/Design

### Study Design

A phase II open-label randomized controlled clinical trial.

### Main Objectives

To assess the efficacy of the following treatments for primary VL at day 28:

• the combination of single dose Liposomal amphotericin B^® ^and a 10 day course of SSG

• the combination of single dose Liposomal amphotericin B^® ^and a 10 day course of Miltefosine

• a 28 day course of Miltefosine

### Setting

The areas of the two trial sites are endemic for VL. They are Dooka Hospital, Gedarif State, Sudan and Kimalel Hospital, Baringo district, Kenya. Addition of another Sudanese site will be considered. Data are brought to the Data Centre based at the study's Coordination Centre in Nairobi, Kenya. The trial is being conducted by the Leishmania East Africa Platform (LEAP: http://www.dndi.org/leap-platform) in collaboration with the trial sites and is sponsored by the Drugs for Neglected Diseases *initiative *(http://www.dndi.org).

### Outcome measures

#### Primary outcome

**Figure 1:**
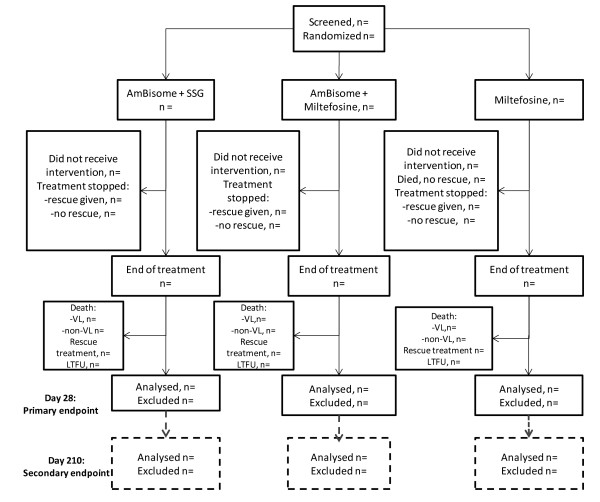
**Consort Trial Diagram**.

The primary endpoint is initial cure, assessed at day 28 (figure 1) based on clinical assessment and parasitologic evaluation (bone marrow or lymph node for Sudan and bone marrow or spleen aspirate for Kenya).

#### Secondary outcomes

• Final cure, defined as the percentage of patients cured at day 210 (6 months follow-up) based on clinical evaluation, with parasitology done only if clinically indicated according to a standardised clinical assessment.

• Adverse events and serious adverse events occurring in the three study arms up to day 60.

• Description of the pharmacodynamic properties of all the three arms.

• Description of the pharmacokinetic properties of Miltefosine alone and in combination with Liposomal amphotericin B^®^.

### Allocation

Randomization sequences were generated, stratified by site in pre-determined block sizes by the Data Centre. Randomization codes have been concealed from investigators at the trial sites using sealed sequentially numbered, opaque envelopes. A copy of the randomization list is securely stored at the Data Centre. The envelopes are numbered sequentially externally. Inside each one there is a randomization sheet with the same sequential number as well as the treatment allocation. These randomization sheets are filed in the source documents which the monitor verifies at each site visit to confirm that only one envelope was opened for each patient to ensure integrity of the randomization process.

### Interventions

Liposomal amphotericin B^® ^is being given as a single dose on day 1 at a dose of 10 mg/kg body weight infused in 5% dextrose running for 1-2 hours.

Miltefosine is being given orally at a dose of 2.5 mg/kg body weight daily, up to a maximum of 150 mg, for 28 days when used alone.

Miltefosine is being given orally at a dose of 2.5 mg/kg body weight daily, up to a maximum of 150 mg, for 10 days starting on day 2 following a single dose of Liposomal amphotericin B^® ^10 mg/kg.

SSG is being given IV/IM at a once daily dose of 20 mg/kg body weight daily for 10 days starting on day 2 following a single dose of Liposomal amphotericin B^®^.

Therefore the dose regimens is as follows

• Liposomal amphotericin B^® ^one dose of 10 mg/kg body weight (IV) on day 1 followed by 10 days of SSG at 20 mg/kg body weight (IV/IM) from days 2-11.

• Liposomal amphotericin B^® ^one dose of 10 mg/kg body weight (IV) on day 1 followed by 10 days of miltefosine at 2.5 mg/kg body weight (oral) from days 2-11.

• Monotherapy course of miltefosine at 2.5 mg/kg body weight (oral) from days 1-28.

All failure with compliance will be documented on the trial medication pages of the CRF.

### Screening

#### Inclusion criteria

Patients with clinical signs of VL (fever for at least 2 weeks and splenomegaly) and diagnosis confirmed by visualization of parasites in tissue samples on microscopy, aged between 7 and 60 years inclusive, signed written informed consent, negative HIV status.

#### Exclusion criteria

Patients who have received any anti-leishmanial drugs in the last 6 months/relapse cases, negative lymph node/bone marrow or spleen smears, severe protein and or caloric malnutrition, previous history of hypersensitivity reaction to SSG or Amphotericin B, suffering from concomitant severe infection such as TB or other serious underlying disease which would preclude evaluation of patients response to study medication, suffering from other conditions associated with splenomegaly such as schistosomiasis, previous history of cardiac arrhythmia or an abnormal ECG, female of child bearing age (pregnant or lactating), haemoglobin <5 mg/dL, WBC <1 × 10^3^/mm^3^, platelets <40,000/mm^3^, abnormal liver function tests (ALT and AST) of more than three times the upper limit of the normal range, serum creatinine outside the normal range for age and gender, major surgical intervention within two weeks prior to enrolment. These tests are standardised but it is not feasible to standardise routine clinical laboratory assessments as different machines and reagents are being used. However both internal and external QC will be carried out at both sites on a regular basis and training offered to site staff at the beginning and during the trial to ensure that the data collected are reliable and comparable.

### HIV-status and VCT

All patients are offered counselling and screening for HIV (voluntary counselling and testing programme (VCT). This is done at the same time as consent is obtained for inclusion in the trial. Patients who decline VCT or are found to be HIV positive are not eligible to participate in this trial but receive appropriate treatment, according to national treatment guidelines. Additionally, they are referred onwards for treatment, surveillance and follow up according to the national protocol for HIV positive patients.

### Consent

Standardised consent forms, adapted to the local context and translated into local languages and approved by ethics committees are being used. Signatures or thumbprints are obtained for consent, with witnesses in the latter case (illiterate subjects) for adults. In the case of children, the investigator gets their assent once permission of either the child's parent or guardian has been obtained.

Patients who do not meet inclusion criteria or who do not give consent are offered free treatment outside the trial, according to national treatment guidelines.

### Analysis and sample size

#### Analysis of primary endpoint: cure at day 28

The study is designed and will be analyzed according to group-sequential methods, specifically the triangular test [10-12]. Following the trials main objectives, the aim of the analysis is to efficiently identify a regimen that is a) inadequate, so its development can be discontinued, or b) adequate, so its development can proceed. In these terms, the highest efficacy (i.e. percentage of patients cured as per primary outcome) considered inadequate is set in this trial to be 75% (denoted p_0_), and the lowest efficacy considered adequate as 90% (denoted p_a_). The null and alternative hypotheses are *H*_0_: *p *≤ *p*_0 _and *H*_1_: *p *>*p*_a _respectively.

**Figure 2:**
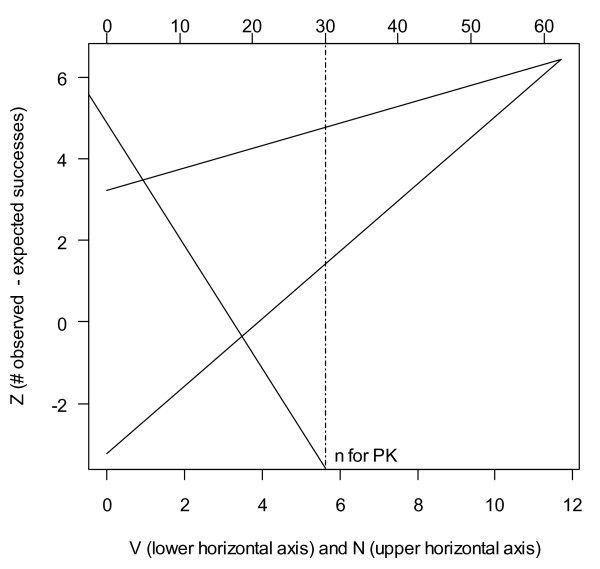
**Triangular region for study arms**. Showing the boundaries for analyzing the sequential trial using the Triangular test with the following parameters (p_0 _= 0.75, p_a _= 0.9, α = 0.05, β = 0.05, and n = 10). The vertical line at *n *= 30 indicates that, for the PK component of the trial, a minimum of 30 patients per arm will be recruited, unless the lower boundary has been crossed before then.

The triangular stopping boundary, as shown in Figure 2, was defined following Ranque et al [10]. With both type I and type II error rates set at 5% (power 95%), and sequential analysis being done after every 15 patients per arm, the maximum sample size required is 189 (63 per arm). The actual sample size may be less than this, depending on when the actual percentage of patients cured leads to a boundary being crossed. The boundaries were calculated using custom-written functions in the R software [13], having checked that they could reproduce the example in Ranque et al [10].

Each analysis consists of calculating the quantities *V *(proportional to current sample size) and Z (number of observed minus expected treatment successes) and plotting them on Figure 2. Hence, for each arm, a line from the origin is plotted over time. Recruitment in an arm will be stopped when this line crosses either boundary of the triangular region. Crossing the lower or upper boundary means concluding inadequacy or adequacy, respectively. Since the three arms will be assessed separately, it is possible that one of them may be stopped prior to others.

The above procedure is subject to a constraint imposed for the PK component of the trial, which requires at least 30 patients in arms 2 and 3, subject to efficacy not being inadequate. Therefore, if the upper boundary is crossed at *n *= 15 (indicating adequate efficacy) that arm will be continued regardless until *n *= 30. However, if the lower boundary is crossed (indicating inadequate efficacy) at *n *= 15, then it will be stopped.

Crossing a boundary of the region implies that the percentage of patients cured is either greater than 90% (if the upper boundary is crossed) or no more than 75% (if the lower boundary is crossed). The maximum likelihood estimate of the efficacy, i.e. the number cured divided by the number of patients, is a biased estimator due to the sequential nature of the trial. To take this into account, the analysis will follow Bellissant et al [11]. First, the sample quantity C* will be calculated as , where V* is the terminal value of *V *from the data sample, and the *θ*_*a *_parameter is the log-odds ratio *θ *= log_e_(*p*(1- *p*_0_)/*p*_0_(1- *p*)) for *p *= *p*_*a*_. Then, the tables in Whitehead's Appendix A [12] will be used to obtain the significance level of the test, and point and interval estimates of *p*. The latter are provided in terms of factors by which to multiply *θ*_*a*_. Finally, these three values of *θ *(i.e. point estimate and upper and lower confidence limits) will be transformed to corresponding sample values of *p *by *p *= (*p*_0_*e*^*θ*^)/(1+ *p*_0_(*e*^*θ*^-1)). Analysis will be done using STATA [14].

#### Analysis of secondary endpoint: cure at day 210

The sequential analysis described above relates only to efficacy on day 28, not day 210. However, because the latter is likely to be highly correlated with the former, ignoring the sequential design in the latter analysis could give inconsistent results. For example, if the status of all patients is the same at day 210 as at day 28, then using different methods for analysis would give different efficacy estimates from the same data. This is because the day 28 estimate takes into account the sequential design which, in general, would differ from conventional analysis done on the same data at day 210.

Therefore, the day 210 efficacy will be estimated by considering it in terms of events over two consecutive time periods: up to day 28, and from day 28 to day 210. More specifically, the day 210 efficacy (i.e. percentage cured or *p*_210_) will be considered as the sum of two probabilities, as follows:

i) probability of being cured at day 28 and remaining cured at day 210.

ii) probability of not being cured at day 28 but becoming cured at day 210.

Each of these probabilities is the product of two terms and enables a point estimate of *p*_210_:

where:

*r *is the percentage of those people cured at day 28 who remain cured at day 210.

*s *is the percentage of those people not cured at day 28 who become cured at day 210.

The confidence interval will be estimated via the sampling variance of *p*_210_. Using the 'delta method' [15], this can be estimated as a function of the sampling variances of *p*_28_, *r *and *s*; these three quantities being statistically independent. The sampling variance of *p*_28 _will be estimated as the square of its standard error, which in turn will be estimated as the width of its 95% confidence interval divided by 2 × 1.96, using a normal approximation. The sampling variances of *r *and *s *will be estimated by considering them as standard binomial proportions.

The above procedure ensures that, for example, if no patients change cure status between day 28 and day 210 then the point estimates of *p*_28 _and *p*_210 _will be equal (because *r *= 1 and *s *= 0). The confidence intervals will, however, generally be unequal. This is because treating the progress between day 28 and day 210 as a separate variable implies additional sampling variation. For example, *r *has sampling variation even when equal to 1. Moreover, sampling variation is considered on the probability scale, not the log-odds scale as for *p*_28_. This also means that the calculated confidence interval could exceed the interval 0-1. In this case, it will be truncated at the limits of that interval.

When the analysis results in an arm being stopped, the final cure rate (day 210) will also be evaluated (when all those patients have reached that time point). If the point estimate of the day 210 cure rate is found to be <90%, then the arm (arm 1 & 2) will not be considered for further study (e.g phase 3).

Within each arm, the percentage cured at each time point will be presented by site. Chi-squared or Fisher's exact tests will be used to test for differences between sites in terms of primary and secondary efficacy endpoints at the 5% level of significance.

### Schedule of Assessments and Expected Side Effects

Assessments are timed at days 0, 3, 7, 14, 21, 28, 60 and 210 (Table 1) and include clinical, parasitology, haematology, biochemistry and pharmacokinetic assessments (table 1). Baseline assessments include anthropometric, clinical and lab evaluations. Day 60 and 210 assessments require some flexibility on dates due to patient travel, visit windows for each is as follows- day 60 (+/- 10 days) and day 210 (+/- 21 days).

**Table 1:** Schedule of events

SCHEDULE OF EVENTS																									
**Protocol Activities and Forms to Be Completed**	**Screening (day)**				**Treatment (day)**																			**At Follow-up after day 30**^1^	
			
			**-2**	**-3**				**4**	**5**	**6**	**7**									**21**					**Day 60 Day 210**

Consent form &																									

											X							X		X		X		X	X

	X																					X			X ^2^

rK39 dipstick	X																								

ECG	X										X							X							

	X										X							X						X	

	X						X				X							X						X	

	X										X							X				X			

	X										X							X				X		X	X

																									

						X	X	X	X	X	X	X	X	X	X										

					X																				

						X	X	X	X	X	X	X	X	X	X										

					X	X	X	X	X	X	X	X	X	X	X	X	X	X		X		X			

**PK study for Miltefosine - Arm 2 - A*dults5***						X^6^		X			X				X									X	X

**PK study for Miltefosine - Arm 2 - *Children5***						X^6^					X				X									X	X

**PK study for Miltefosine - Arm 3 - A*dults5***					X^6^		X				X							X		X		X		X	X

**PK study for Miltefosine - Arm 3 - *Children5***					X^6^						X							X				X		X	X

Volume of blood (ml) taken from Group 2 - children	7.5					2.5	2.5				10.5				0.5			10				7.5		7.5	7.5

Volume of blood (ml) taken from Group 3 - children	7.5				2.5		2.5				10.5							10.5				8		7.5	7.5

^1 ^Patients assessment between day 60 and 210 in the event of any medical problems. Blood count, biochemistry, liver function tests parasitology and PK analysis will be done in the event of such a visit. At the day 210 visit, flexibility (of +/- 30 days) will be allowed for timing due to practical difficulties patients may face to meet exact timing of visits.

^2 ^Parasitology will be done in the follow up period after day 28 if clinically indicated (i.e. if reappearance of symptoms and signs of VL).

^3 ^2.5 ml of EDTA blood for Complete blood count; and only 0.2 ml EDTA blood used for PCR

^4 ^7.5 ml blood for urea, creatinine, liver function test.

^5 ^The PK study will be done on the specified days with 2.5 ml of EDTA blood taken before/after and 0.5 ml during treatment (the possible use of dried blood spots is currently under investigation).

^6 ^On first day of Miltefosine treatment, blood samples will be drawn 3 times: just prior to first dose, 4 & 8 hours post-dose to assess the absorption-phase of miltefosine (total volume needed 2.5 ml).

Volumes of blood required for children are shown in bottom two rows. In total, 41.5 ml are required in the first 1 month during admission in group 3 and 41 ml for group 2.

Clinical assessments will include pulse, blood pressure, temperature, weight, height (done only at baseline), spleen size, liver size.

### Rescue medication

The decision to give rescue medication is based on a standard guideline for all trial sites. Any patient who receives rescue medication is considered a treatment failure at initial cure if receipt of rescue occurs on or prior to initial cure (day 28) and treatment failure at final cure if it occurs on or prior to final cure (day 210).

Rescue treatment to be given includes:

- Liposomal amphotericin B 30 mg/kg IV split into multiple doses (according to country protocol: Sudan - 3 mg/kg/day for 10 days)

- SSG 20 mg/kg IM for 30-60+ days: for patients not responding to initial rescue treatment or for patients requiring treatment for severe PKDL.

### Post Kala-azar Dermal Leishmaniasis (PKDL)

A complication of Visceral Leishmaniasis, particularly in Sudan is post-Kala-azar Dermal Leishmaniasis (PKDL). PKDL is characterised by skin lesions which normally occur in the months following treatment in people who have recovered from VL. Patients are being monitored closely for PKDL at days 0, 28, 60 and 210. Diagnosis will be made clinically, based on the typical appearance and distribution of the rash.

### Concomitant Medication

Concomitant medication necessary for the health of the patient is permitted during the course of the study. Details of all concomitant medication taken during the study are recorded in the CRF with indication, daily dose, route and dates of administration.

### Ancillary Studies

#### Pharmacokinetics

The effects of VL disease and geographical differences on the pharmacokinetics (PK) of miltefosine remain largely unknown, with most available PK data coming from a relatively healthy European patient group with cutaneous leishmaniasis (CL) [16]. A thorough PK study of miltefosine in adult VL patients has not been published, let alone in paediatric patients and no such data are available from the East-African region. This study will describe the pharmacokinetic profile of miltefosine in adults and children (>7 years of age) in both the monotherapy and combination therapy arm. Any significant PK interactions between liposomal amphotericin B and miltefosine will be assessed in the combination treatment arm.

Bioanalysis of miltefosine will be done on plasma samples with a volume of 50 to 250 μL taken during both treatment and follow-up (see table 1), using a validated liquid chromatography tandem mass spectrometry (LC-MS/MS) assay for miltefosine [17]. In children, a more sparse sampling strategy will be applied because of ethical concerns.

The PK of miltefosine will be modelled and analyzed using a population PK approach, which enables, for example the estimation of within- and between-subject variations. Non-linear mixed-effects modelling will be performed using the NONMEM statistical software package [18]. The minimal value of the objective function (equal to minus twice the log likelihood) provided by NONMEM will be used as a goodness-of-fit characteristic, in addition to comparisons of standard errors of parameter estimates. Furthermore, performance of the models will be assessed via standard goodness-of-fit plots. Covariate models for body size will be evaluated stepwise to explain possible differences between paediatric and adult patients, including allometric or linear scaling of clearance and volume of distribution by either body weight or fat-free mass. The appropriateness of the covariate model will be evaluated by comparison of the objective function value given by NONMEM and resulting reduction in unexplained between-subject variability of the respective pharmacokinetic variables.

#### Pharmacodynamics

To assess treatment response during and after treatment, this study will apply repeated measurements of blood parasite loads using a real time reverse transcriptase polymerase chain reaction (qPCR) for *L. donovani *in EDTA blood based on the amplification of single-stranded 18S rRNA as a pharmacodynamic (PD) marker [19,20]. Differences in parasite clearance in the blood are to be expected between the treatment arms: combinations are likely to result in a more rapid elimination of *Leishmania *parasites, hence the decreased duration of treatment. In this trial the blood parasite counts will be used as a PD marker of the parasite clearance rate and thus response to treatment.

In the arms receiving miltefosine, the outcome and parasite clearance measured by qPCR will be linked to miltefosine PK. Modelling of miltefosine PK/PD in our patients will enable us to establish variability in pharmacokinetics in relation to outcome, which is an essential component in the development of new treatment regimens.

### Training

All trial site staff; physicians, nurses, laboratory technicians and pharmacists received training on the study protocol, study specific procedures and International conference on harmonisation-good clinical practise (ICH-GCP) guidelines [21,22]. Additional training sessions will be provided as required, using external consultants where necessary. Documentation of receipt of training is maintained at the Coordination Centre.

### Quality control and quality assurance

Suitably qualified Clinical Monitors trained in GCP regularly visit trial sites to monitor all aspects of the trial including; informed consent procedures, drug accountability, source data verification, adverse event reporting, sample handling, analysis and secure data storage.

### Data collection and data management

Data are to be recorded on 3-part no carbon required (NCR) case report forms (CRFs) by site investigators, transcribed from hospital source data. Unique patient identifiers, assigned at the time of randomisation, are linked to unique hospital record numbers. During monitoring visits, CRF data are cross-checked against hospital source data as much as possible by the monitor. The top sheet for each page of the CRF is brought to the central Data Centre for double-entry into GCP compliant open-access database software OpenClinica, version 2.7 [23] with range checks implemented to detect unusual values at data entry, before analysis. Following validation, data will be read into Stata, version 11 special edition [14] and pre-programmed command files will be used to generate lists of data value queries in a thorough data cross-checking process. Query forms are to be automatically generated via Microsoft Access^© ^database software and emailed to trial sites, copied to clinical monitors. The sites will print and make resolutions. At the next monitoring visit, monitors will verify resolutions and deliver to the Data Centre. Data corrections will be programmed in Stata to complete the data cleaning audit trail. At analysis, any unusual values detected would be verified with the investigator at the sites before final analysis is done to confirm correctness/completeness. This will also be captured in the data management report prior to database lock.

### Publication policy

DNDi, as sponsor, will render all necessary assistance to investigators to ensure timely publication of results in an international peer-reviewed, for the benefit of patients and to inform national decision-making on VL treatment guidelines. Ancillary studies will acknowledge those involved by name where appropriate.

### Confidentiality

Trial site records will contain names and residential information for each patient to allow follow-up to take place. Only the unique numeric identifier assigned to patients will be extracted from patient records and transferred to the Data Centre. Patient data will be kept securely at trial sites under the responsibility of the site investigator.

### Audit

During the course of the trial, site audits will be undertaken to assess compliance with ICH-GCP guidelines. Specific issues to be assessed include adherence to the protocol and standard operational procedures (SOPs), consent, laboratory practise, documentation and record keeping. All areas of non-compliance will be addressed by the site investigators or trial coordination centre depending on the findings.

### Termination of the study

On approaching the end of planned recruitment, the trial coordination office will send written instructions by email to each site regarding the date to cease recruitment. All patients will be followed up as per the protocol, with the data being collected and cleaned. Once the data lock has been completed, site close out visits will be performed by the coordination team and clinical monitors. A decision for premature termination will be taken in consultation and agreement with the sponsor, investigators and the DSMB. All relevant ethics committees and regulatory authorities will also be informed of the reason for termination. Trial master files and completed CRFs will be archived by the coordination centre in Nairobi for 15 years.

### Dissemination of Results

After recruiting 15 patients per arm, a brief interim analysis report presenting the efficacy, including the triangular analysis will be provided to the Data Safety Monitoring Board (DSMB) whose composition is based on WHO-TDR guidelines [24]. The DSMB membership includes a pharmacologist, paediatrician and an epidemiologist. The DSMB will seek advice from an external statistician when necessary. This report will also contain a listing of serious adverse events occurring prior to the analysis date. Adverse event listings are to be provided at the request of the DSMB. The DSMB, through the Chair may request additional efficacy and safety data if they have concerns relating to trial conduct or other ethical and safety issues.

At the end of the trial, the clinical study report will be circulated to Principal Investigators, DSMB, Ethics Committees and Ministries of Health.

### Ethical Approval

Ethical approval has been obtained from National and local Ethics Committees in Kenya and Sudan prior to the start of the trial in each Country. Ethical approval has also been granted by LSHTM Ethics Committee, the AMC Medical Ethics Committee issued a 'declaration of no objection'.

### Organisation

The DNDi Coordination Team, based mainly at the Coordination Centre, DNDi Africa, Kenya Medical Research Institute, Nairobi, are responsible for collation and submission of protocol amendments, organisation of training for trial staff, monitoring and supervision of trial conduct, day to day management of the trial, harvesting data collected at each site, trial monitoring visits and data management, all to GCP standards.

## Discussion

Due to limitations of current treatments, and the risk of drug resistance developing, there is an urgent need to develop short course combination treatments against VL in the East Africa region. The aim of this trial is to evaluate potential combinations that can then be evaluated in a large multi centre phase-III clinical trial in the region. The current trial also aims to collect additional data to assist in the registration of miltefosine in the region. For this reason, three separate arms with no between-arm comparisons were chosen and a PK/PD component focusing on the miltefosine treatments included. Due to potential geographical differences in drug response within the region, the trial aims to evaluate treatment in two sites: one from the northern part (Sudan) and one from the Southern part (Kenya) of the East African foci [3]. To make efficiencies in development, cost and time, the study design used the triangular test. This also meant that the primary endpoint of day 28 was selected, since a long follow up time precludes the optimal use of such triangular tests. The design also used the experience of a phase-II trial of combinations conducted in India [25]. Important potential outcomes of this study include not only the registration of miltefosine in the region, and the identification of drug combinations for phase III development, but also the validation of a design that could be used in the drug development process of future new chemical entities against VL.

## List of Abbreviations

LEAP: Leishmaniasis East Africa Platform; IM: Intramuscular; IV: Intravenous; PK: Pharmacokinetics; PD: Pharmacodynamics; VL: Visceral Leishmaniasis; RCT: Randomised controlled trial; SSG: Sodium stibogluconate; PKDL: Post kala-azar dermal leishmaniasis; DSMB: Data safety monitoring board; CRF: Case report form; ICH: International Conference on Harmonisation; GCP: Good Clinical Practise; SOP: Standard Operating Procedure; NCR: No Carbon Required; VCT: Voluntary Counselling and Testing.

## Competing interests

DNDi, as sponsor, is funding the trial, costs relating to maintenance of the LEAP platform and open-access publication of LEAP Study Group manuscripts. MW, RO, SE and MB are current employees of DNDi.

## Authors' contributions

All authors have read and approved the final manuscript.

LEAP Study Group Investigators (MW, EAGK and AM), statisticians from LSHTM (NA, TE) and representatives from the sponsor, DNDi, (MB, SE) designed the study. RO, NA and TE drafted this submission.

All other LEAP study group authors played an important role in finalising the study protocol.
